# Supplementary material for: Nanostructure Engineering via Intramolecular Construction of Carbon Nitride as Efficient Photocatalyst for CO2 Reduction
Source: Nanomaterials (Basel). 2021 Nov 29;11(12):3245. doi: 10.3390/nano11123245 (PMC8706010; doi:10.3390/nano11123245)
Supplement: Supplementary file 1 [file nanomaterials-11-03245-s001.zip › nanomaterials-1445044-supplementary.pdf]

Supplementary Materials

Nanostructure Engineering via Intramolecular Construction of Carbon Nitride as Efficient Photocatalyst for CO_2 Reduction

Muhammad Sohail [1], Tariq Altalhi [2], Abdullah G. Al-Sehemi [3], Taha Abdel Mohaymen Taha [4,5], Karam S. El-Nasser [6,7], Ahmed A. Al-Ghamdi [8], Mahnoor Boukhari [9], Arkom Palamanit [10], Asif Hayat [11,*], Mohammed A. Amin [2,*] and Wan Izhan Nawawi Bin Wan Ismail [12,*]

1 Yangtze Delta Region Institute (Huzhou), University of Electronic Science and Technology of China, Huzhou 313001, China; sohailncp@gmail.com
2 Department of Chemistry, College of Science, Taif University, P.O. Box 11099, Taif 21944, Saudi Arabia; ta.altalhi@tu.edu.sa
3 Department of Chemistry, Faculty of Science, Research Center for Advanced Materials Science (RCAMS), King Khalid University, P.O. Box 9004, Abha 61413, Saudi Arabia; agsehemi@kku.edu.sa
4 Physics department, College of Science, Jouf University, Sakaka 75471, Saudi Arabia; themaida@ju.edu.sa
5 Physics and Engineering Mathematics Department, Faculty of Electronic Engineering, Menoufia University, Menouf 32952, Egypt
6 Chemistry Department, College of Science and Arts, Jouf University, Al-Gurayyat 77447, Saudi Arabia; karamsaif@ju.edu.sa
7 Chemistry Department, Faculty of Science, Al-Azhar University, Assiut 71524, Egypt
8 Department of Physics, Faculty of Science, King Abdulaziz University, Jeddah 21589, Saudi Arabia; agamdi@kau.edu.sa
9 College of Material Science & Engineering, Beijing University of Technology, Beijing 100081, China; bukharimahnoor11@gmail.com
10 Energy Technology Program, Department of Specialized Engineering, Faculty of Engineering, Prince of Songkla University, 15 Karnjanavanich Rd., Hat Yai, Songkhla 90110, Thailand; energy_man001@hotmail.com
11 State Key Laboratory of Photocatalysis on Energy and Environment, College of Chemistry, Fuzhou University, Fuzhou 350116, China
12 Faculty of Applied Sciences, Universiti Teknologi MARA, Cawangan Perlis, Arau Perlis 02600, Malaysia
* Correspondence: asifncp11@yahoo.com (A.H.); Mohamed@tu.edu.sa (M.A.A.); wi_nawawi@uitm.edu.my (W.I.N.)

1. Chemicals

All the chemicals used for photocatalytic experiments were of high grade and used directly without any further purification. Chemicals such as Urea, 1,4-dihydroxybenzene (DHB), 2,2–bi-pyridine (bpy), $CoCl_2$ were obtained from Sigma-Aldrich. The organic solvents such as dimethyl sulfoxide (DMSO), N,N-dimethylformamide (DMF), acetonitrile (MeCN) and tetrahydrofuran (THF) were received from China Sinopharm Chemical Reagent Co. Ltd. During CO_2 reduction, carbon dioxide gas was used are of high purity (99.999%) and were obtained from Fuzhou Lianzhong Industrial Gases Co., Ltd. Similarly, the ^{13}CO was supplied from Beijing Hess chemical gas industry.

1.1. Characterizations

Samples were characterized by various techniques and hence the powder X-ray diffraction (XRD) of our samples was carried out on a Bruker D8 Advance diffract meter with Cu Kα1 radiation. Similarly, a Nicolet Magna 670 Fourier transform infrared spectrometer was used for getting the Fourier transform infrared spectroscopy (FT-IR) data for our samples. The Brunauer–Emmett–Teller (BET) surface area N_2 adsorption-desorption and CO_2 adsorption isotherms were characterized by using Micrometrics ASAP2020 equipment. The samples were conducted to check its morphologies by using Hitachi New Generation SU8010 field emission scanning electron microscope (FESEM). A JEOL model JEM 2010 EX instrument was used to obtain the transmission electron microscopy (TEM) images of as-prepared samples. X-ray photoelectron spectroscopy (XPS) measurements were carried out on Thermo ESCALAB250 instrument with a

monochromatic Al Ka line source (200 W). A Bruker Advance III 500 spectrometer for the characterization of the solid-state 13C NMR spectra of our samples. The electron paramagnetic resonance (EPR) measurements were conducted both under light and dark area on a Bruker model A300 spectrometer connected with a 300 W Xe lamp equipped having a UV-cutoff (> 420 nm) used as visible light source. The UV-vis diffuse reflectance spectra (DRS) data of the samples were obtained by using a Varian Cary 500 Scan UV–vis spectrophotometer and similarly Photoluminescence (PL) spectra were conducted on an Edinburgh FI/FSTCSPC 920 spectrophotometer. The samples were photo catalytically used for CO_2 reduction by using an Agilent 7820A gas chromatograph having thermal conductivity detector (TCD) and a TD-01 packed column for produced gases detection simultaneously. A HP 5973 gas chromatography-mass spectrometry (GC–MS) were used for several $^{13}CO_2$ isotopic experiment and hence ^{13}CO generating from the $^{13}CO_2$ respectively. The surface potentials of the electrodes were measured using kelvin probe force microscopy (KPFM). To measure the difference in work function between the sample and the tip, a Bruker Dimension V SPM system with Pt/Ir coated tips (resonant frequency: 72 kHz) was used. Surface potentials are measured in the dark and under monochromatic light irradiation, respectively.

2. Photocatalytic CO_2 Reduction

Typically, CO_2 reduction reaction was performed photo catalytically in a schlenk flask two mouth reactors, having 80 ml of aggregate capacity. The flask reactor contains 30 mg of catalyst by weight, 1 μmol of $CoCl_2$, 15 mg of 2,2-bipyridine as a photosensitizer, 1 ml of triethanolamine (TEOA) and 6 ml of solvent i.e., 5 ml of acetonitrile (MeCN) and 1 ml of water (H_2O). The reactor was kept continuously for stirring and air pump was connected for vacuum degassing for almost 3 times on one side of reactor flask and the other mouth portion was connected with pure CO_2 containing balloon for back filling. Similarly, when adsorption-desorption equilibrium maintains after the evacuation of air, then keep the reactor under Xe lamp of 300 W having 420 nm cut-off filter. In the whole process, the temperature of the system was controlled at 30 °C by connecting the cooling water machine to the system. The schlenk flask was kept 15 cm away from the xeon arc lamp. The produced gases after the photocatalytic irradiation were injected to an Agilent 7820A gas chromatography connected to the system. Under same condition, several control experiments were evaluated using $^{13}CO_2$ as the reactant by reduced into ^{13}CO that were analyzed by Gas chromatography-mass spectrometry (GC-MS).

2.1. Photocatalytic Degradation of RhB Dye

The photocatalytic degradation was performed under visible light using Xe lamp 300 W. The photocatalyst well performed the degradation of dye using (Rhodamine B) RhB in the whole experiment. Typically, 20 mg of photocatalyst fine powder was dispersed in a Pyrex glass photo-reactor containing 80 ml of RhB red color aqueous solution under continuous stirring at room temperature by connecting circulating water machine to the system. The aqueous solution was maintained to be stirred magnetically for half an hour in order to stabilize adsorption-desorption equilibrium. After monitoring the equilibrium state, a Xe 300 W lamp having 420 nm cut off filter was turned on in front of photo-reactor suspension used as a photocatalytic illumination source respectively. The analytical samples were collected from the main solution at a fixed interval of time and keep continue this process until the photocatalytic degradation process complete. After withdrawing, all the collected samples were centrifuged to remove the solid catalyst particles for analysis. A UV-Vis spectrophotometer was used for analyzed the concentration of suspension after centrifugation and measures its absorbance by calculating Lambert-Beer law equation. Similarly, pure RhB without photocatalyst was also collected during experiment and evaluate its photo activity under desired condition.

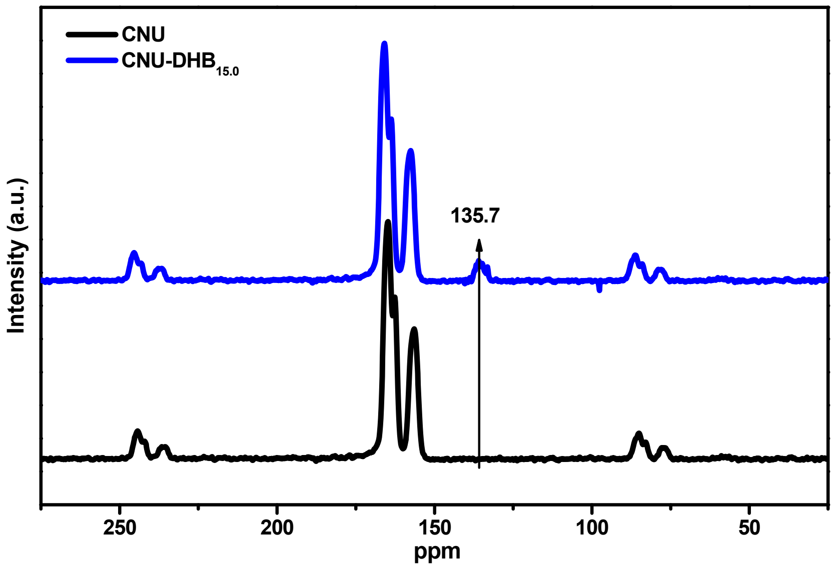

Figure S1. Solid-state ^{13}C NMR spectra of pure CNU and CNU-DHB15.0 samples.

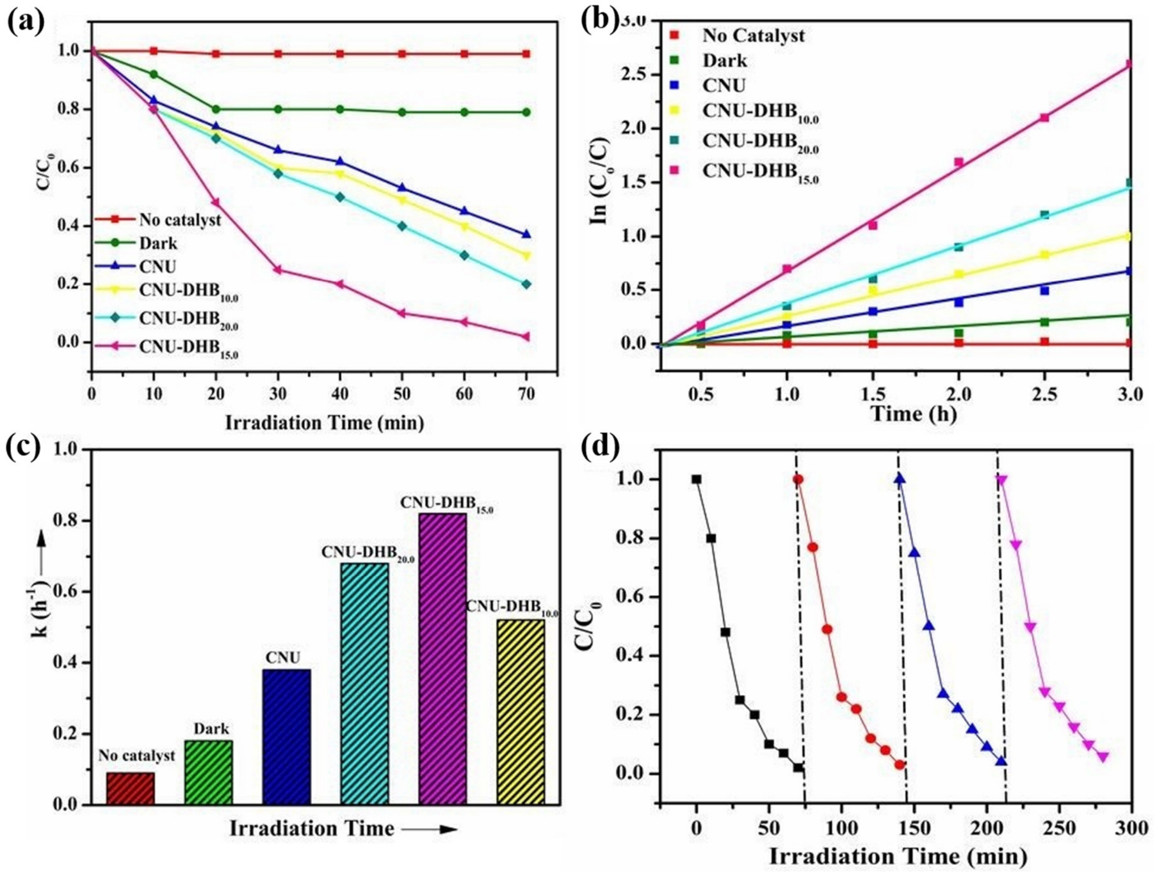

Figure S2. (a) Visible light photocatalytic degradation **(b)** first-order kinetics data **(c)** the apparent rate constants for RhB degradation **(d)** Cycling runs of CNU-DHB15.0 catalyst for the photocatalytic degradation of rhodamine (RhB) dye (λ = 420 nm).

Table S1. comparison of photocatalytic performance of CO_2 reduction.

Precursor	Monomer	Copolymerized Catalyst	Photocatalytic CO_2 Reduction	References
Urea	Porphyrin monomer	CN-TDP	24.3 μmol/h^{-1}	[1]
Urea	Polyethyleneimine	u-XPEI	10.3 μmol/h^{-1}	[2]
Urea	Barbituric acid	CNU-BA	30.9 μmol/h^{-1}	[3]
Urea	Trimesic acid	CNU-TMA	31.2 μmol/h^{-1}	[4]
Urea	dibromopyridazine	CNU-DBP	25.3μmol/h^{-1}	[5]
Urea	2,5-dibromothiophene	UCN-TP	27.9 μmol/h^{-1}	[6]
Urea	dibromopyridazine	CNU-DAP	21.6μmol/h^{-1}	[7]
Urea	dihydroxy benzene	CNU-DHB	32.5 μmol/h^{-1}	This work

Table S2. Different parameters of our synthesized photocatalysts.

Catalyst	SA[a] (m^2/g)	C/N ratio	Band gap (eV)	CO/H_2 evolution μmol/h^{-1}
CNU	49.9	63	2.89	3.2/0.71
CNU-$DHB_{5.0}$	99.7	68	2.57	17.4/6.1
CNU-$DHB_{10.0}$	107.5	0.69	2.31	27.2/5.9
CNU-$DHB_{15.0}$	123.7	0.71	2.25	32.5/6.4
CNU-$DHB_{20.0}$	101.3	0.69	2.49	24.7/5.8

Reaction conditions for CO_2 reduction: 30 mg of photocatalyst dispersed in solvent (MeCN)/H_2O = 5:1) containing $Co(bpy)_3Cl_2{\cdot}6H_2O$ as a photosensitizer and triethanolamine (TEOA) as a sacrificial electron donor, 300 W Xe-lamp, λ>420 nm. [a] Surface Area.

References

1. Hayat, A., et al., A Superficial Intramolecular Alignment of Carbon Nitride through Conjugated Monomer for Optimized Photocatalytic CO_2 Reduction. Catalysts, 2021. **11**(8): p. 935.

2. Song, X., et al., Enhanced light utilization efficiency and fast charge transfer for excellent CO_2 photoreduction activity by constructing defect structures in carbon nitride. Journal of Colloid and Interface Science, 2020. **578**: p. 574–583.

3. Qin, J., et al., Photocatalytic reduction of CO_2 by graphitic carbon nitride polymers derived from urea and barbituric acid. Applied Catalysis B: Environmental, 2015. **179**: p. 1–8.

4. Hayat, A., et al., Synthesis and optimization of the trimesic acid modified polymeric carbon nitride for enhanced photocatalytic reduction of CO_2. Journal of colloid and interface science, 2019. **548**: p. 197–205.

5. Hayat, A., et al., Organic Conjugation of Polymeric Carbon Nitride for Improved Photocatalytic CO_2 Conversion and H_2 Fixation. Energy Technology, 2021: p. 2100091.

6. Hayat, A., et al., A butterfly shaped organic heterojunction photocatalyst for effective photocatalytic CO_2 reduction. CrystEngComm, 2021. **23**(28): p. 4963–4974.

7. Hayat, A., et al., π-deficient pyridine ring-incorporated carbon nitride polymers for photocatalytic H_2 evolution and CO_2 fixation. Research on Chemical Intermediates, 2021. **47**(1): p. 15–27.
